# Effects of 12 weeks of Nordic Walking and XCO Walking training on the endurance capacity of older adults

**DOI:** 10.1186/s11556-017-0186-2

**Published:** 2017-09-12

**Authors:** Tobias Morat, Jenny Krueger, Angus Gaedtke, Manuela Preuss, Joachim Latsch, Hans-Georg Predel

**Affiliations:** 1 Institute of Movement and Sport Gerontology, German Sport University Cologne, Am Sportpark Muengersdorf 6, 50933 Cologne, Germany; 2 Institute of Cardiology and Sports Medicine, German Sport University Cologne, Am Sportpark Muengersdorf 6, 50933 Cologne, Germany; 3 Present Address: Healthy Campus Bonn, Department 10: Personnel Progress & Career, Rhenish Friedrich-Wilhelms University Bonn, Walter-Flex-Str. 3, 53113 Bonn, Germany

**Keywords:** Walking, Endurance training, Intervention, Elderly, Seniors, Oxygen uptake, Endurance capacity, Aging, Physical activity

## Abstract

**Background:**

Several studies have already examined the positive effects of various forms of endurance training in patient groups and in healthy adults up to 60 years old. The aim of this study was to analyse the effects of Nordic Walking (NW) and XCO Walking (XCO) training on endurance capacity in healthy older adults, aged 60 years and older.

**Methods:**

Twenty-three older participants (mean age: 69.9 ± 5.4 years) were randomly assigned to either the NW group or the XCO group. All participants were measured before and after the 12 weeks of endurance training (2 sessions/week) to examine oxygen uptake (VO_2_) and energy consumption during an outdoor field test. In addition, heart rates were recorded and lactate samples were collected.

**Results:**

NW mainly demonstrated some significant (*p* < 0.05) decreases in heart rate, lactate concentration at lower to moderate walking speeds, whereas XCO Walking revealed significant (*p* < 0.05) decreases in lactate concentration and VO_2_ at low to higher walking speeds.

**Conclusions:**

NW as well as XCO training increase the efficiency of the cardio-vascular system in older subjects. Both training approaches are suitable options for endurance training, which may serve to counteract age- and inactivity-related decreases in cardio-vascular functioning as well as aid in maintaining overall performance in older adults.

## Background

With increasing age, endurance capacity declines and can lead to functional problems in everyday life [1–6]. Several studies in recent years have used Nordic Walking as the means of choice to achieve positive effects on the cardiovascular and musculoskeletal system because of the higher activity in the muscles involved in the Nordic Walking (NW) technique [4, 7–10].

For NW, several studies with different patient groups (aged 40 years and older) have demonstrated positive effects on oxygen uptake, perceived exertion [11, 12], the anaerobic threshold [13–17] and the reduction of body fat [17, 18]. However, the former studies with NW varied in their study design (cross-sectional, longitudinal including an intervention), setting (laboratory, field), walking area (treadmill, walking track), test protocol (increasing speed, elevation of pitch angle, distance, duration), and technical instructions [19–26]. In addition, most of the NW studies had measurements in laboratory set-ups, although NW is an outdoor endurance training form. For example, two intervention studies with NW were conducted for 50 min, three times a week, for 6 weeks [20] and 40 min, four times a week, for 13 weeks [22]. Chomiuk et al. [20] showed, for example, higher oxygen uptake values after their NW training intervention with patients over 65 years of age compared to an inactive control group. Kukkonen-Harjula et al. [22] compared Walking (W) with and without poles and demonstrated similar reductions of heart rates and lactate concentrations in both groups with women aged 50 to 60 years. Preuss et al. [27] compared NW, W, Power Walking and Jogging in 32 healthy persons with a mean age of 46 years. The authors showed significant higher values in heart rate and relative oxygen uptake during NW, compared with W. As a possible explanation a more difficult arm-leg coordination during NW was assumed [27]. Furthermore, the former NW studies with field tests were cross-sectional in their design and varied extensively in their testing and training protocols [27–32]. A current review [9] gives a comprehensive overview of the impact of Nordic Walking in the second half of lifetime and displays the literature concerning the effectiveness and safety of Nordic Walking in the therapeutic rehabilitation of patients of an advanced age. Another review [10] highlighted the health benefits of NW as well. Within the reviews, the authors concluded that heart rate and oxygen uptake values were higher when compared with W [9, 10].

Similar effects on the cardiovascular system are attributed to XCO Walking (XCO), another endurance training alternative [33]. For XCO Walking, participants use two XCO trainers (a single XCO trainer is a 27 cm long closed aluminium tube filled with movable slate granules, fixed weight: 630–650 g). Participants grab one XCO trainer with each hand and move them alternating back and forth during normal walking. With each swing to the front and back the slate granules hit the end caps of the XCO trainers, resulting in the XCO-typical “reactive impacts”, producing a reflexive activation of antagonist shoulder muscles [34].

At present, there are just two studies that examined potential effects of XCO Walking. Two cross-sectional studies have looked at the muscle activity of the biceps and triceps, but not during the usual XCO Walking technique [35], and have compared heart rate, oxygen uptake and energy consumption during the usage of the XCO trainers versus dumbbells [36]. Only one study has implemented a longitudinal design with an intervention period [33]. These authors [33] compared XCO Walking with walking without any tool in untrained women (40–60 years). Unfortunately, the XCO group trained with the interval endurance method and the walking group with the continuous endurance method. Due to this flaw, it is not obvious whether the effects are a result of using the XCO trainers or because of the different training methods.

In summary, for NW, there are results available for adults and several patient groups, but for XCO, the research knowledge is very limited. Both the NW and XCO studies solely included healthy adults up to 60 years old or specific patient groups; however, the two endurance training alternatives, NW and XCO, could be reasonable ways to positively influence the endurance capacity of older adults aged 60 years and older, as well as to prevent cardiovascular diseases, for example. It is questionable whether these two different technique models, with the dynamic activation of m.biceps brachii and m.triceps brachii during NW, on the one hand, and the isometric activation of these two muscles during XCO, on the other hand, lead to similar effects on the endurance capacity of older adults.

The aim of this study was to examine the physiological effects of two endurance training alternatives (NW versus XCO) on the endurance capacity of healthy older adults (aged 60 years and older).

## Methods

### Study design

The study was conducted as a randomised controlled longitudinal trial with two parallel training groups (NW and XCO). Both groups completed 4 weeks of technical instruction training followed by a 12-week endurance training. The main assessments were measured prior to (T1) and after (T2) the 12 weeks of endurance training. The participants were randomised either to the NW group using Nordic Walking poles or to the XCO group using XCO trainers. Both groups obtained 4 weeks of technical instruction training (twice a week for 60 min) with their specific training tool (NW poles or XCO trainers) prior to T1 to get familiar with the correct technique and handling of their specific device. In advance of their participation, all of the participants were fully informed about the purpose and experimental procedures of the study. All of the participants completed consent forms. The participants were informed that all data collected would be processed anonymously. The study was conducted in the City of Cologne. The study was conducted according to the Declaration of Helsinki. The ethics committee at the German Sport University Cologne granted ethical approval.

### Participants

Twenty-three older adults (9 men, 14 women) with a mean age of 69.9 ± 5.4 years (mean body height: 1.68 ± 0.1 m; mean body mass: 77.3 ± 17.5 kg) participated. The participants were recruited by advertisements placed on web pages and in local newspapers, and with flyers in Cologne. Detailed eligibility was checked via a health questionnaire. Furthermore, the physicians of potential participants had to provide them with a medical clearance certificate confirming that none of the following exclusion criteria were present (cardiovascular disease, acute infections, renal or hepatic problems, thrombophlebitis, disc prolapse during the last year, unstable diabetes, neurological and neuromuscular diseases, arterial hypertension, diagnosed gait disorders, artificial joints, osteoporosis, need of walking aids). Any experience in systematic NW or XCO Walking within the past 12 months was also a given exclusion criteria. After the completion of the study, all participants obtained the possibility to continue physical activity (endurance training) in a local cooperating sports club.

### Intervention

The first intervention phase consisted of 4 weeks of technical instruction training (TIT; see Table 3 in the Appendix) and 12 weeks of endurance training (ET; see Table 4 in the Appendix), 2 sessions a week with at least 1 day of rest in between sessions (Tuesday and Thursday). Each session lasted 60 min. The NW group trained 16 weeks with NW poles, and the XCO group with XCO® trainers (FLEXI-SPORTS GmbH, Munich, Germany). At the beginning of each session, there was 5–10 min of warm-up, which was followed by 40–45 min of the main part of the training with the specific content and 5–10 min of cool-down. TIT was implemented to ensure the correct techniques of all of the participants and to reduce bias in the endurance field-testing results because of uneconomic techniques [27, 31]. For ET, heart rate (HR) percentage areas were individually prescribed for each participant (based on their peak HR during the stress test = 100%). The individual peak heart rate was taken from the exercise electrocardiogram during a defined stress test on a cycling ergometer, which was done by the physicians of the participants. The peak HR represents the highest HR that was observed during the stress test. Thus, training intensities should be interpreted in that context. All of the participants were equipped with POLAR® HR belts and RS400 monitors (POLAR, Kempele, Finland) during all ET sessions to control and monitor their HR. Training intensity was progressively increased over the 12 weeks of ET (see Table 4 in the Appendix). At first, they started with 60% of their peak HR, this was progressively increased ending with 85%.

### Measurements

Oxygen uptake (VO_2_) and energy consumption were measured with a portable indirect calorimetric device (MetaMax 3B®, Cortex, Germany) during an outdoor lactate step test with progressively increasing walking speeds. Every participant performed the test with the assigned training tool, either NW poles or XCO trainers, on a typical 400 m track and field running track with five progressively increasing speeds (1.0 m/s, 1.2 m/s, 1.4 m/s, 1.6 m/s 1.8 m/s, 5 min per speed, 3 min rest between stages). In addition, their heart rates were recorded with POLAR HR belts and RS400 monitors (POLAR, Kempele, Finland) and lactate samples were collected. The dependent primary outcome variables were oxygen uptake [ml/min], energy expenditure [kcal], lactate concentration [mmol/l] and heart rate [beats/min].

The German Physical Activity Questionnaire 50^+^ [37] was used to examine physical activity, leisure time and social activities as a secondary outcome. During all ET sessions, the HR of the participants was recorded with the POLAR HR belts and RS400 monitors (POLAR, Kempele, Finland); distance, walking speed and the area of training were measured through Global Positioning Data (GPS) as it was done in former studies [38, 39]. Therefore, an Android Smartphone Galaxy SII (Samsung, Suwon, South Korea) and the SensorLog application (version 1.6; Bernd Thomas) was used.

### Statistical analysis

All data are presented as mean (*M*) ± standard deviation (*SD*). All statistics were analysed using the Statistical Package for the Social Sciences (version 23.0; IBM SPSS, Chicago, IL). Within this study, an intention-to-treat analysis with the data of 23 cases was performed. Missing data were analysed with the MCAR-Test (missing completely at random) by Little. Afterwards, a multiple imputation for monotone missing data with 15 imputations [40, 41] was conducted with SPSS to maintain a complete dataset of all 23 randomised cases. With the Shapiro-Wilk test, the normal distribution of the data was inspected statistically. The baseline characteristics of the sample were compared with an independent T-test for normally distributed variables and a Mann-Whitney-U-Test for all others and non-parametric variables. Mauchly’s sphericity test and Levene’s test (if necessary including a Lilliefors correction) were used to test the sphericity and homogeneity of the variance assumptions, respectively. A two-factor repeated-measures analysis of variance (ANOVA), as a mixed design (general linear model) with the effect of factor one (walking speed stage) and factor two (measurement session), was used. In the case of no sphericity, the Greenhouse-Geisser correction was used. If the ANOVA displayed significant effects, the estimated marginal means (EMMEANS) with Bonferroni correction were used (post hoc) to identify the specific significant differences between the individual walking speed stages or measurement sessions (T1 and T2). Eta squared (*η* ^*2*^) was calculated to evaluate small (*η* ^*2*^ = 0.02), middle (*η* ^*2*^ = 0.13) or large (*η* ^*2*^ = 0.26) effects. An alpha <0.05 was considered as statistically significant.

## Results

The characteristics of the sample displayed no significant differences between the two groups, NW and XCO, which was indicative of a successful randomisation (see Table 1).

**Table 1:** Characteristics (means (M) ± standard deviations (SD)) and *p*-values of the sample, separated by group (Nordic Walking = NW, XCO Walking = XCO)

Variables	NW group	XCO group	*p*-value
Number of participants	*n* = 12	*n* = 11	
Gender	5 males, 7 females	4 males, 7 females	
Age [years]	69.9 ± 5.4	69.2 ± 8.1	0.80
Body height [m]	1.67 ± 0.10	1.69 ± 0.10	0.63
Body mass [kg]	79.0 ± 18.3	75.6 ± 16.7	0.65
German PAQ 50^+^ score	11,120 ± 3324	13,937 ± 1280	0.67

### Training data

The mean values for heart rate during training sessions displayed no significant differences (*p* = 0.73) between groups. In Table 2, all relative heart rates for both training groups NW and XCO are presented. Taking all 24 training sessions into account, the NW group trained with a mean relative heart rate of 73 ± 4% and the XCO group with 75 ± 3%.

**Table 2:** Relative target and achieved heart rates for the NW and XCO groups in the 24 training sessions

Session	Relative target heart rate	Achieved heart rate NW	Achieved heart rate XCO
1	60–75%	68%	76%
2	60–75%	69%	72%
3	60–75%	71%	70%
4	60–75%	67%	73%
5	60–75%	68%	66%
6	70–80%	72%	79%
7	70–80%	79%	79%
8	70–80%	75%	79%
9	70–80%	77%	77%
10	70–80%	74%	78%
11	75–85%	72%	76%
12	75–85%	68%	74%
13	75–85%	71%	72%
14	75–85%	71%	76%
15	75–85%	73%	74%
16	80–85%	76%	76%
17	80–85%	73%	75%
18	80–85%	74%	77%
19	80–85%	72%	77%
20	80–85%	73%	78%
21	80–85%	77%	78%
22	80–85%	79%	77%
23	80–85%	78%	76%
24	80–85%	79%	77%

An analysis of the GPS data showed neither a significant difference for total distance (*p* = 0.95; NW: 4.96 km; XCO: 4.94 km) nor for the mean walking speed between minute 20 to minute 40 (main part) of the training sessions (*p* = 0.93; NW: 6.51 km/h; XCO: 6.49 km/h) between groups (NW and XCO). All routes within the training sessions covered an area in the locality of 4 km.

**Fig. 1:**
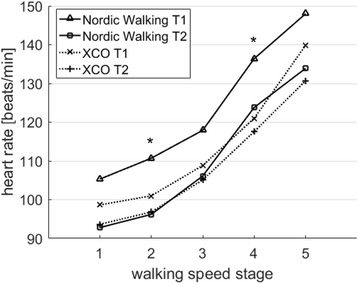
Means of heart rate [beats/min] in the NW group and the XCO group before (T1) and after (T2) the 12-week endurance training during the 5 walking speed stages (stage 1: 1.0 m/s; stage 2: 1.2 m/s; stage 3: 1.4 m/s; stage 4: 1.6 m/s; stage 5: 1.6 m/s); * = significant difference (*p* < 0.05) between T1 and T2 in the NW group; # = significant difference (*p* < 0.05) between T1 and T2 in the XCO group

Regarding heart rate, there was a significant (*p* < 0.05) effect for the measurement session (*p* < 0.05) and the walking speed stage. The NW group displayed a mean heart rate reduction of 5% between T1 and T2, which was significant (*p* < 0.05) at stages 2 and 4. The XCO group revealed no significant effect of the measurement session (see Fig. 1).

For lactate concentration results, the ANOVA detected a significant (*p* < 0.05) effect of the measurement session and a significant (*p* < 0.05) effect for the walking speed stage at T1 and T2 for the NW group. The NW group showed a mean reduction of 32% in lactate concentration through the training with significant differences (*p* < 0.01) at stages 1 to 3.

**Fig. 2:**
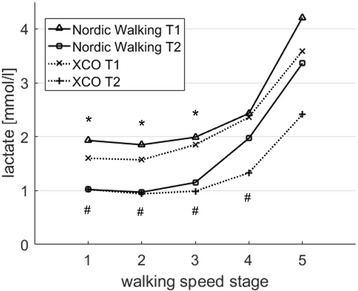
Means of lactate concentration [mmol/l] in the NW group and the XCO group before (T1) and after (T2) the 12-week endurance training during the 5 walking speed stages (stage 1: 1.0 m/s; stage 2: 1.2 m/s; stage 3: 1.4 m/s; stage 4: 1.6 m/s; stage 5: 1.6 m/s); *significant difference (*p* < 0.05) between T1 and T2 in the NW group; # = significant difference (*p* < 0.05) between T1 and T2 in the XCO group

The XCO group showed a significant measurement session effect (*p* < 0.01) and a significant walking speed stage effect (*p* < 0.05) as well. The XCO group decreased their mean lactate concentration by about 40% with significant (*p* < 0.01) reductions for stages 1 to 4 (see Fig. 2).

**Fig. 3:**
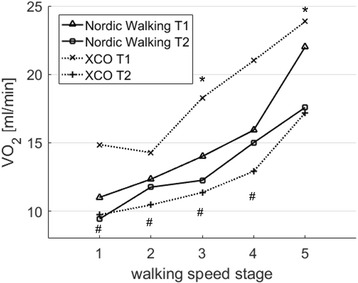
Means of VO_2_ in the NW group and the XCO group before (T1) and after (T2) the 12-week endurance training during the 5 walking speed stages (stage 1: 1.0 m/s; stage 2: 1.2 m/s; stage 3: 1.4 m/s; stage 4: 1.6 m/s; stage 5: 1.6 m/s); VO_2_ = oxygen uptake in [ml/min]; *significant difference (*p* < 0.05) between T1 and T2 in the NW group; # = significant difference (*p* < 0.05) between T1 and T2 in the XCO group

There was a significant effect of the measurement session (*p* < 0.01) for the oxygen uptake (VO_2_) and of the walking speed stage (*p* < 0.01) in the NW group. On average, through all 5 stages, the VO_2_ of the NW group was 12% lower after the training. Post hoc tests showed a significantly (*p* < 0.05) lower VO_2_ at stage 3 and at stage 5 at T2 compared to T1 (see Fig. 3).

The XCO group showed a significant effect of the measurement session (*p* < 0.01) for VO_2_ and of the walking speed stage (*p* < 0.01) as well. The XCO group displayed a reduction in VO_2_ through the training between T1 and T2 by 33% with significant (*p* < 0.05) differences for stages 1 to 4 (see Fig. 3).

Energy consumption displayed a significant effect of the measurement session (*p* < 0.05) and a significant effect of the walking speed stage (*p* < 0.05) for the NW group. The mean energy consumption for the NW group was reduced by 15% through the training, post hoc tests showed a significant difference (p < 0.05) for stage 1 between T1 and T2. The XCO group showed the same significant (*p* < 0.05) ANOVA results for both factors. A significant (*p* < 0.01) difference between T1 and T2 was present for stage 4, and the mean reduction of energy consumption was 15% in the XCO group.

All means before (T1) and after (T2) the 12 weeks of training and eta squared of the tests are displayed in Table 5 in the Appendix. Significant (*p* < 0.05) post hoc differences for heart rate, lactate concentration oxygen uptake and energy consumption between individual walking speed stages for the NW group and the XCO group are presented in the Tables 6 and 7 in the Appendix.

## Discussion

The absence of significant differences regarding the participants’ characteristics is indicative of a successful randomisation to either the NW or the XCO group prior to the first training session.

The NW group showed decreased heart rate, lactate concentration and energy consumption with the same walking speeds at T1 and T2 after the training period, particularly at slower to moderate walking speeds. Further, the NW group decreased their VO_2_ in moderate to high walking speeds. The XCO group displayed no significant differences for heart rate for the comparison before and after the training period. The XCO group demonstrated decreases in lactate concentration and VO_2_ in slow to high walking speeds (with the exception of 1.8 m/s). The reductions in heart rate in the NW group and lactate concentrations in both groups through the training period can be interpreted as an improved cardiovascular function or as a response to a reduced metabolic demand. Regarding VO_2_, the XCO group revealed higher average (33%) decreases than the NW group (12%). However, our study was not conducted in a crossover design and both groups completed the outdoor field endurance test before and after the 12 weeks of training only with their assigned training tool (XCO trainer or NW poles). Therefore, a direct comparison between the two training groups is not possible.

In this study, the increase in the metabolic demand was given by the speed of each walking speed stage. With this in mind, the change (decrease) in VO_2_ after the training period during the same walking speed can be interpreted as a change of efficiency in the older adults. Usually, this can be the case due to the technical component of this type of exercise although all participants have been taught within 8 sessions and realized the correct technique. If the training resulted in improved efficiency (decreased VO_2_), the reduction in lactate concentration and heart rate could possibly be explained as a response to the reduction in the metabolic demand for the exercise. The positive changes in the measured parameters imply that both training tools were effective for maintaining (improving) endurance capacity in healthy older adults (60 years and older). To further study these aspects, an identical exhaustive stress test without the training tools (possibly standardized on a treadmill) before and after the training period can provide further insights.

A study by Kukkonen-Harjula and colleagues [22] demonstrated improvements in both the NW (2.5 ml/min/kg) and the Walking (W) group (2.6 ml/min/kg) in peak VO_2_, energy consumption and lactate concentration after 13 weeks of NW and W. However, their sample included women up to 60 years old, their study included a testing protocol for submaximal and maximal exercise performance with both NW and W for all participants, and the authors provided only two instruction sessions on NW and W. However, authors did not find statistically significant differences between the study groups in the submaximal performance tests and no significant interaction [22]. Other considerations concerning NW have been predominantly in cross-sectional designs in the laboratory and in the field as well [21, 23, 27–29, 31]. These author groups demonstrated the advantages of NW in oxygen uptake, energy consumption, lactate concentration and heart rate in comparison with normal walking. For XCO, there are only a few studies, for example, by Stengel and colleagues [33], who showed improved oxygen uptake after their intervention with the XCO trainers. These authors [33] compared XCO Walking with normal walking; however, a main problem with their study and a limitation of its relevance is the application of two different training methods in the two groups: Walking (W) and XCO Walking (XCO). The W group trained with the continuous endurance method, whereas the XCO group received interval training. Due to this flaw, one could not conclude whether the effects resulted from the different training tools or the endurance methods. Additionally, their participants were 29 untrained women between 40 and 60 years of age. The same author group displayed higher cardio-pulmonary, as well as metabolic stress (oxygen uptake, energy consumption, heart rate) in participants using the XCO trainers in contrast to using dumbbells with fixed weights [36].

Altogether, comparisons between the studies mentioned are difficult due to differences in the studies’ design, samples, measurement methods and interventions, and should be taken as additions that lead to a more comprehensive picture of different ways to improve or maintain endurance capacity, particularly for the target group of older adults aged 60 years and older. This study provides relevant results regarding the two training tools, Nordic Walking poles and XCO trainers, to improve endurance capacity in older adults.

The advantages of this study are the identical days and times-of-day of the (parallel) training sessions, the same instructors, and the same training methods, walking speeds and distances. Furthermore, this study consisted of 4 weeks of technical instruction training and 12 weeks of endurance training, resulting in a total intervention duration of 16 weeks. Compared with other studies [20, 22, 33] in this field, it is one of the longest intervention periods. The intervention design of this study is similar to the recommendations by Skorkowska-Telichowska et al. in their review, who suggest NW with professional trainers 2–3 times a week over at least 3 months. Based on their review, these authors conclude that the positive training effects can be maintained over 6–9 months after the training ends [9].

Our study examined the effects of NW and XCO training on the endurance capacity. With the everyday life of older adults in mind, positive effects through NW and XCO training on the functional capacity of older adults can also be an important aspect. For example, Parkatti et al. [4] observed positive effects of NW and Walking on different functional tests with significant differences compared to an inactive control group. These authors had 40 older adults (mean age of 69 years) doing 9 weeks with two times week and 60 min per session. However, these authors merged their walking and NW groups to one intervention group and no further statements regarding the two forms NW versus Walking can be made [4].

## Conclusions

In conclusion, both Nordic Walking and XCO Walking are suitable endurance training alternatives for older adults (60 years and older) to positively effect heart rate, lactate concentration and oxygen uptake as parameters of endurance capacity. Following 8 technical instruction sessions, the participants of both training groups reached similar decreases in these parameters by realising training intensities between 60 and 85% of their individual peak heart rates in 24 training sessions in the locality of 2 km from the starting point. Through the training with both NW poles and XCO trainers, these changes can be interpreted as a change of efficiency. This study is one of the first to provide the (long-term: 12 weeks) results of identical and systematic endurance training using Nordic Walking poles and XCO trainers in persons aged 60 years and older. Both NW and XCO are suitable endurance training alternatives to positively influence the efficiency of the cardiovascular system and can result in maintained performance to counteract age-related degradation processes.

## Appendix

**Table 3:** Technical training sessions and their contents (same for NW and XCO)

Session	Content
1	Introduction of the basic technique (make participants familiar with the device, walking and standing exercises)
2	Repetition and expansion of the basic technique (repetition of basic elements, partner/group exercises)
3	Technique expansion (reflexion and discussion of technique faults, technique correction by partners)
4	Technique practice (preparation for different terrains, technique practice with various tasks and conditions)
5	Technique stabilisation (repetition of the most important technique elements)
6	Technique practice with different velocities
7	Technique practice with different terrains
8	Technique stabilisation

**Table 4:** Endurance training sessions with their training intensity, methods and duration (same for NW and XCO)

Session	Training intensity [% of peak heart rate]	Training method (duration)
1–5	60–75%	Extensive continuous method (40 min)
6–10	70–80%	Extensive continuous or extensive interval method (40 min)
11–15	75–85%	Intensive continuous or extensive/intensive interval method (45 min)
16–24	80–85%	Intensive continuous or intensive interval method (45 min)

**Table 5:** Means ± standard deviation (SD), differences with the 95% confidence intervals (CI) and eta squared (η^2^) of heart rate, lactate, VO_2_, and energy consumption during the outdoor lactate step test before (T1) and after (T2) the 12 weeks of Nordic Walking (NW) or XCO Walking (XCO) training

	Before (T1), means (SD)	After (T2), means (SD)	Difference (95% CI)	η^2^
Heart rate [beats/min]				0.03
NW				
1.0 m/s	105.3 ± 18.1	92.8 ± 12.1	−12.5 (−27.0–2.0)	
1.2 m/s	110.7 ± 11.2	96.2 ± 11.7	−14.5 (−30.2–1.2)	
1.4 m/s	117.9 ± 9.1	106.1 ± 10.2	−11.8 (−26.2–2.5)	
1.6 m/s	136.4 ± 9.2	123.8 ± 12.8	−12.6 (−22.5 – −2.7)	
1.8 m/s	148.0 ± 9.6	133.9 ± 12.3	−14.7 (−19.3–4.2)	
XCO				
1.0 m/s	98.6 ± 9.1	93.6 ± 9.3	−5.0 (−12.5–2.5)	
1.2 m/s	101.0 ± 21.3	96.8 ± 9.1	−4.2 (−14.7–6.4)	
1.4 m/s	108.8 ± 22.1	105.1 ± 10.7	−3.7 (−18.0–10.4)	
1.6 m/s	121.0 ± 16.3	117.6 ± 14.5	−3.5 (−12.4–5.5)	
1.8 m/s	139.9 ± 23.8	130.7 ± 22.4	−3.7 (−13.3–5.9)	
lactate [mmol/l]				0.07
NW				
1.0 m/s	1.93 ± 0.55	1.02 ± 0.40	−0.50 (−0.73 – −0.27)	
1.2 m/s	1.85 ± 0.36	0.97 ± 0.40	−0.61 (−0.84 – −0.39)	
1.4 m/s	1.99 ± 0.34	1.15 ± 0.50	−0.94 (−1.39 – −0.49)	
1.6 m/s	2.43 ± 0.52	1.97 ± 1.10	−1.17 (−1.89 – −0.44)	
1.8 m/s	4.21 ± 1.28	3.36 ± 2.03	−0.68 (−2.70 – −0.12)	
XCO				
1.0 m/s	1.60 ± 0.42	1.02 ± 0.32	−0.58 (−0.89 – −0.27)	
1.2 m/s	1.57 ± 0.25	0.94 ± 0.26	−0.62 (−0.78 – −0.45)	
1.4 m/s	1.85 ± 0.36	0.99 ± 0.30	−0.86 (−1.10 – −0.60)	
1.6 m/s	2.36 ± 0.63	1.33 ± 0.46	−1.03 (−1.45 – −0.61)	
1.8 m/s	3.59 ± 1.72	2.41 ± 1.28	−1.01 (−2.60–0.50)	
VO_2_ [ml/min]				0.02
NW				
1.0 m/s	11.0 ± 3.6	9.4 ± 2.5	−1.8 (−4.7–1.0)	
1.2 m/s	12.3 ± 2.9	11.8 ± 2.5	−2.3 (−6.0–1.3)	
1.4 m/s	14.0 ± 3.5	12.3 ± 2.6	−2.8 (−5.2 – −0.5)	
1.6 m/s	15.9 ± 1.0	15.0 ± 2.9	−1.4 (−4.2–1.4)	
1.8 m/s	22.0 ± 5.6	17.6 ± 2.8	−2.0 (−4.7–0.9)	
XCO				
1.0 m/s	14.9 ± 4.3	9.7 ± 2.6	−3.8 (−7.2 – −0.4)	
1.2 m/s	14.3 ± 3.1	10.5 ± 2.6	−3.3 (−6.4 – −0.2)	
1.4 m/s	18.3 ± 2.8	11.4 ± 2.9	−4.9 (−8.8 – −1.0)	
1.6 m/s	21.0 ± 4.2	12.9 ± 4.3	−5.3 (−9.7 – −0.8)	
1.8 m/s	23.9 ± 3.8	17.2 ± 6.5	−3.9 (−7.8–0.1)	
Energy consumption [kcal]				0.02
NW				
1.0 m/s	30.0 ± 7.7	25.7 ± 11.4	−4.3 (−11.6–2.9)	
1.2 m/s	32.6 ± 6.0	28.3 ± 6.8	0.1 (−9.4–9.6)	
1.4 m/s	36.6 ± 4.1	34.7 ± 8.8	−1.9 (−14.7–10.8)	
1.6 m/s	38.7 ± 10.4	36.4 ± 11.3	−4.2 (−23.8–15.5)	
1.8 m/s	86.1 ± 40.6	52.2 ± 7.4	−10.0 (−102.3–82.3)	
XCO				
1.0 m/s	36.0 ± 8.8	28.3 ± 6.9	−7.7 (−12.3 – −3.2)	
1.2 m/s	36.0 ± 9.0	27.8 ± 7.5	−15.9 (−26.7 – −5.2)	
1.4 m/s	38.7 ± 8.4	29.9 ± 6.8	−24.7 (−41.2 – −8.2)	
1.6 m/s	42.9 ± 7.7	34.6 ± 8.4	−33.0 (−53.5 – −12.5)	
1.8 m/s	49.1 ± 16.5	39.4 ± 11.5	−41.1 (−58.3 – −24.0)	

**Table 6:** Significant differences (* = *p* < 0.05) of the comparisons between the means at individual walking speed stages (st) at the two measurement sessions T1 (before the training) and T2 (after the training) for the Nordic Walking group for the tested parameters heart rate (= hr), lactate concentration (= lc), oxygen uptake (= VO_2_) and energy consumption (= ec)

st		2				3				4				5			
		hr	lc	VO_2_	ec	hr	lc	VO_2_	ec	hr	lc	VO_2_	ec	hr	lc	VO_2_	ec
1	T1									*			*	*	*	*	*
	T2	*		*		*	*	*		*	*	*		*	*	*	*
2	T1								*				*	*	*	*	*
	T2					*			*	*	*	*	*	*	*	*	*
3	T1												*		*	*	*
	T2									*	*	*		*		*	*
4	T1													*	*		*
	T2													*	*		*

**Table 7:** Significant differences (* = *p* < 0.05) of the comparisons between the means at individual walking speed stages (st) at the two measurement sessions T1 (before the training) and T2 (after the training) for the XCO Walking group for the tested parameters heart rate (= hr), lactate concentration (= lc), oxygen uptake (= VO_2_) and energy consumption (= ec)

st		2				3				4				5			
		hr	lc	VO_2_	ec	hr	lc	VO_2_	ec	hr	lc	VO_2_	ec	hr	lc	VO_2_	ec
1	T1				*			*	*	*	*	*	*	*	*	*	
	T2	*				*		*		*		*	*	*	*	*	*
2	T1					*	*	*	*	*	*	*	*	*	*	*	
	T2					*			*	*		*	*	*	*	*	*
3	T1									*	*		*	*	*	*	
	T2									*				*	*	*	
4	T1													*	*		
	T2													*	*	*	

## Abbreviations

ANOVA: Analysis of variance
CI: Confidence interval
EMG: Electromyography
EMMEANS: Estimated marginal means
ET: Endurance training
GPS: Global Positioning Data
HR: Heart rate
IL: Illinois
M: Mean
m.: Musculus
MCAR: Missing completely at random
NW: Nordic Walking
SD: Standard deviation
SPSS: Statistical Package for the Social Sciences
T1: Measurement session 1
T2: Measurement session 2
TIT: Technical instruction training
VO2: Oxygen uptake
W: Walking
XCO: XCO Walking
